# The influence of SYRIZA-ANEL Greek health policies on hospital efficiency

**DOI:** 10.1186/s12961-023-01032-3

**Published:** 2023-08-22

**Authors:** Georgios I. Farantos, Nikitas-Spiros Koutsoukis

**Affiliations:** University of Peloponnese, 1 Aristotelous & Leof. Athinon, Corinth, Greece

**Keywords:** Health policies, Inputs, Outputs, Data Envelopment Analysis, SYRIZA-ANEL government

## Abstract

**Background:**

We analyse the impact of the three following categories of Health Policies (HP) carried out by the Greek SYRIZA-ANEL governments on the efficiency of Greek public general hospitals. These governments have implemented policies intended to change the rate of contributions to publicly funded healthcare (PCnH), policies to affect the volume and quality of publicly funded health care (PVQH) and those intended to affect the costs of publicly funded healthcare (PCH). A literary review of the PCnH. PVQH and PCH policies of the Greek SYRIZA-ANEL governments was carried out and an efficiency window-DEA study was executed using data from the Ministry of Health (MoH) and the Greek Statistical Authority (ELSTAT).

**Methods:**

The study was designed to assess the impact of PCnH. PVQH and PCH policies by the Greek SYRIZA-ANEL governments on the efficiency of Greek general hospitals. The data was collected from HEAL-Link scientific journals. Information on HPs was extracted from the work collected. The values of inputs and outputs used for the efficiency study were obtained from ELSTAT and Greek MoH databases.

**Results:**

HPs of the Greek SYRIZA-ANEL governments extend to all three HP categories of the sample used. These policies have a dual effect on both the inputs and outputs used in efficiency. Efficiency values exhibit fluctuations with good and bad years. The SYRIZA-ANEL governments seek to ensure more equality in access to health services. Some of the policies reduce costs and have a positive impact on efficiency, while others have the opposite effect. The increase in outputs achieved as a result of health policies is counter balanced by an increase in inputs.

**Conclusions:**

The PCnH, PVQH and PCH policies of the SYRIZA-ANEL governance seem to have a dual orientation: some policies reduce the cost of a category and contain the total cost, thus positively contributing to an increase in efficiency. Certain policies are aimed more at fulfilling the criterion of equality in the provision of health services and thus the cost inevitably increases. From the window-DEA study, three relatively “good” years emerge (2015, 2016, 2018) and two “bad years” (2017, 2019). This analysis will be useful for further research on the effect of health policies on hospital efficiency in other countries and periods.

## Introduction

HPs exercised by governments affect the inputs or outputs of health units and thus efficiency [1–3]. The aims of HPs vary among different governments, namely: some governments try to develop the patient care system on a community level, take for example the home rehabilitation program for stroke patients [4]. Some try to deal with diseases through human development policies in the health sector [5], while others try to control and eradicate disease by creating disease-free zones, compiling accurate information and applying comprehensive strategies to accelerate disease management [6]. Greek HPs influence the inputs and outputs selected for a hospital efficiency study and a change in efficiency values of Greek hospitals [7]. During an economic crisis (such as the one in which the SYRIZA-ANEL government ruled until 2018), HPs are distinguished according to Kaitelidou and Kouli [8] into policies intended to change the level of contributions to publicly funded healthcare (PCnH), policies intended to affect the volume and quality of publicly funded health care (PVQH) and policies intended to affect the costs of publicly funded healthcare (PCH). These policies seek to increase efficiency by reducing inputs and increasing outputs.

PCnHs such as health budget protection, financial restructuring of hospital sectors and reduction of coverage by introducing or increasing user charges can reduce state health funding [9] and thus increase efficiency. PVQHs (positive or negative) could be a change in population coverage (positive or negative), a change in access to services and a change in service coverage [10] which also affect efficiency. PCHs such as drug price cuts, the introduction of a positive drug list and the introduction of a profit-limiting system in the event of unexpected trade can increase efficiency [11].

Each of the PCnH, PVQH and PCH policies exercised by governments affect the inputs or outputs of health units and consequently their efficiency. Economic funding policies with measures such as performance volume limit financing, or the application of costing through DRG, were observed to reduce hospitalisation days and achieve a large cost saving [12].The extension of health services can increase the number of outgoing patients and thereby efficiency [13]. The development of eHealth reduces the financial costs of health institutions [14, 15]. The introduction of Primary Health Care (PFY) and the management of outpatients affects the efficiency of hospitals [16]. Pharmaceutical cost containment policies, such as pricing, reimbursement, market entry and cost control, can directly reduce hospital costs [17]. Health Technology Assessment (HTA) provides opportunities for information on clinical and political decision-making through systematic evaluation, including, inter alia, a cost-effectiveness method for economic evaluation and treatment selection [18]. Control and containment of hospital supplies contributes to cost containment and cost savings [19].

Data Envelopment Analysis (DEA) is the prevailing method for assessing the efficiency of the healthcare system [20, 21]. The DEA method was originally developed by Farell [22] to measure the change to efficiency of related decision making units [23–25]. Charnes et al. [26] then developed the CCR model which is currently used to measure technical efficiency [27, 28]. Banker et al. [29] introduced the BCC model, which is currently used to measure pure (technical) efficiency [30, 31]. The three efficiency values, technical efficiency (TE), pure (technical) efficiency (PE) and scale efficiency (SE) are related. Charnes et al. [32] introduced an improved method, the window-DEA, which is more suitable for measuring efficiency over extended periods of time.

Measurement of efficiency using the DEA method is carried out using the inputs and outputs involved in the conversion process in the Decision Making Unit (DMU). TE is calculated on the basis of a complex calculation between the change in outputs of each decision making unit versus its inputs [22]. Limiting inputs causes an increase in efficiency [33]. Thus, HP and health sector reforms attempt to reduce inputs in order to increase efficiency. Often, the aim is to keep outputs stable, while inputs decrease (inputs-oriented efficiency). This pursuit exists at a time of budgetary constraints and drastic cost limitation [34].

DEA efficiency studies use DMUs inputs and outputs to measure efficiency. DEA efficiency studies in Greek hospitals were reviewed and the inputs and outputs used in these were extracted. The results of this analysis are shown in Table 1. In spite of the vast number of studies measuring economic efficiency in health care, there has been little follow-up to date by policy-makers [35].

**Table 1:** Selection of most common inputs–outputs in efficiency studies of Greek health units

Researchers	Year	Inputs					Outputs	
		Beds	Doctors	Nursing staff	Administrative personnel	Financial details	Hospitalisation days	Number of patients
Athanassopoulos, Gounaris, and Sissouras	1999		X	X	X	X	X	
Kontodimopoulos, Nanos and Niakas	2006	X	X	X			X	
Aletras, Kontodimopoulos, Zagouldoudis and Niakas	2007	X	X		X			X
Katharaki	2008	Χ	Χ			Χ	Χ	Χ
Tsekouras, Papathanassopoulos, Kounetas and Pappies	2010	X	X	X			X	
Haloes and Tzeremes	2011	X	X	X			X	
Polyzoa	2012	X	X	X	X	X		X
Dimas, Goula and Soulis	2012	X				X	X	
Mitropoulos, Mitropoulos and Sissouras	2013		X	X	X	X		X
Mitropoulos, Mitropoulos and Giannikos	2013		X	X				
Balamatsis and Chondrocoukis	2014	X	X	X	X	X		X
Mitropoulos, Talias and Mitropoulos	2015	X	X		X	X		X
Kaitelidou, Katharaki, Kalogeropoulou, Economou, Siskou, Souliotis, … and Liaropoulos	2016	X	X	X	X	X		X
Oikonomou et al.	2016		X	X				X
Fragkiadakis, Doumpos, Zopounidis and Germain	2016	X	X	X		X		X
Xeons et al.	2017	X	X	X	X	X		X
Takaki’s, Nektarios, Tziaferi and Prezerakos	2021		X	X	X			X

HP decision makers are primarily the Greek Government and the Minister of Health. The managers of Health Regions (YPE) and those of general hospitals are authorised to decide on the application of a HP. Interventions apply to the secondary general hospitals of the whole country without exception. The HP interventions of the SYRIZA-ANEL government start in 2015 and are implemented over the duration of their governance.

### Research motivation

The motivation for the research stems from the desire to study the effect of health policies on efficiency for a given period. This effect has not been studied by other researchers in the past. The results of the research will contribute to postdoctoral research, and they will prove useful in shaping an integrated way of thinking about the impact of HP on efficiency.

### Paper purpose

The purpose of this paper is to analyse the impact of PCnH, PVQH and PCH policies of the Greek SYRIZA-ANEL government on the efficiency of Greek public general hospitals.

### Issue under discussion

More specifically, through our research, we aim to demonstrate how the PCnH, PVQH and PCH policies of the afore-mentioned governments alter the efficiency of Greek general hospitals in the period under study. We suppose that PCnH, PVQH and PCH policies increase the efficiency of the general hospitals. This supposition arises from the fact that the bibliography refers to governmental policies which aim at increasing hospital performance. In fact, this increase has been calculated in certain instances [36, 37]. The impact of the economic crisis on the hospital sector and the efficiency of Greek public hospitals is noted by Kaitelidou et al. [36]. The innovative nature of this particular study is that no similar research appears in the literature review during the period in question.

What is the problem this paper attempts to answer? The problem is how PCnH, PVQH and PCH policies implemented by the SYRIZA-ANEL governments affect the efficiency of Greek public general hospitals.

### Review question

How do the PCnH, PVQH and PCH policies implemented by the SYRIZA-ANEL governments affect the efficiency of Greek public general hospitals?

### Secondary review questions

How do PCnH, PVQH and PCH policies influence the inputs and outputs selected for the Greek general hospitals efficiency study?

How do the efficiency values of Greek hospitals change?

### Main hypothesis of the study Hο

PCnH, PVQH and PCH policies implemented by the SYRIZA-ANEL governments positively influence the efficiency of Greek public hospitals by reducing inputs and increasing outputs.

### Secondary hypothesis of the study H1

PCnH, PVQH and PCH policies of the SYRIZA-ANEL governments reduce inputs and increase outputs.

### Secondary hypothesis of the study Η2

TE changes constantly in a positive way over the period considered.

### Conclusions if the case is verified

Ιf the main hypothesis is verified, this will mean that PCnH, PVQH and PCH policies implemented by the SYRIZA-ANEL governments positively influence the efficiency of Greek public hospitals by reducing inputs and increasing outputs.

### Conclusions if the case is not verified

If the main hypothesis is not verified, this will mean that PCnH, PVQH and PCH policies implemented by the SYRIZA-ANEL governments do not positively influence the efficiency of Greek public hospitals by reducing inputs and increasing outputs.

## Research method

The HEAL-Link Database, which refers to international scientific journals as well as other databases (SCOPUS), was employed to identify the work used in this research. The literary review concerned 6275 papers referring to HPs and health unit efficiency studies. In terms of PCnH, PVQH and PCH policies, 61 papers were used, while in terms of health efficiency, 28 papers were used.

The key words that we used to study HPs were Health Policies, SYRIZA-ANEL government.

Papers which contained an inadequate description of a HP, or those unable to clearly classify policy measures into model categories, were excluded from our study.

### Research protocol

Τhe model of the Center of Reviews and Dissemination (2009) PICOS is used [38].

### Path of the study

This study is a combination of a quantitative one (using the window-DEA method) and a qualitative one (the effect of health policies on the study’s inputs and outputs). Requirements for qualitative and quantitative research are covered by the scope and quality of these studies. PCnH, PVQH and PCH policies during the SYRIZA-ANEL governance consist of the interventions made which influence the inputs and outputs of this research. We also observe that those elements influence efficiency in the long-term. The implementation of each PCnH, PVQH and PCH policy activates a mechanism which influences each input or output of this research. Thus, there is a logical connection between individual policies and their effect on each of the inputs and outputs.

### Data

Data (inputs and outputs) generated by ELSTAT and the Greek MoH is used for the measuring of efficiency. The data is organised into groups in the 7 YPE (regional health sectors) of the country, so that they can be adequately compared. A researcher can find more information and supplementary material in the databases of the Greek Ministry of Health (MoH) (https://www.moh.gov.gr/).

### Research methods

Τhe DEA method was chosen to measure efficiency over the period considered. We studied the most frequently used inputs and outputs from the literature. We have constructed the model of the study presented in Table 2.

**Table 2:** The inputs and outputs used in the study (Authors, 2022)

Inputs/outputs	Name	Description	Exogenous	Weights
Input code				
I	Total purchases	Total amount of purchases	Νο	1
I2	Number of Doctors	The number of doctors of all specialties (excluding paramedics)	No	1
I3	Number of nurses	Number of all nurses	No	0.9
Ι4	Number of administrative staff	Number of all administrative staff	No	0.5
Ι5	Number of beds	The number of deployed beds	No	1
Output code				
Ο1	Patients examined	Number of persons examined in the ERs	Yes	0.9
Ο2	Number of outgoing patients	Number of patients discharged (hospitalised)	Yes	1
Ο3	Hospitalisation days	The sum of the days over which any short or permanent hospitalisation took place	Νο	0.9

The values of TE, PE and SE are measured. Based on the literary review conducted, we select the inputs and outputs that will be used in the model of this study. The study model, which is very detailed, determines the extraction of results. The inputs and outputs of the study, with their codes, name, description, existence of exogenous or non-exogenous character and their weights are shown in Table 2.

The results of the calculation of TE, PE and SE values are extracted with the help of the Hoger Scheel free EMS software.

Then we ranked health policies using a model that was already known. We analysed how PCnH, PVQH and PCH policies impact inflows and outflows. We explained the impact of PCnH, PVQH and PCH policies on efficiency.

### Reporting

The reporting guideline TIDieR-PHP for population health and policy interventions has been used.

### Interventions and comparators

PCnH, PVQH and PCH policies exercised by the Greek SYRIZA-ANEL governments in order to change the financing, cost or health care of the Greek ESY.

### Outcomes

Changes in the efficiency values of Greek general hospitals were measured with the window-DEA method. These changes are explained as a result of the impact of the PCnH, PVQH and PCH policies of the Greek SYRIZA-ANEL governments.

### Study design

PCnH, PVQH and PCH policies of SYRIZA-ANEL governments are investigated and their effects on inputs and outputs of Greek general hospitals are studied. The change in the efficiency of Greek hospitals is measured and interpreted based on the effect of PCnH, PVQH and PCH policies.

In the present study avoidance of bias has been ensured through objectivity by using literary references for each argument.

## Results and discussion

### Results

The results of the calculation of the values of TE, PE and SE which are extracted with the help of the Hoger Scheel free EMS software are shown in Table 3.

**Table 3:** Change in the technical, pure and scale efficiency of Greek general hospitals during the study period

	2015	2016	2017	2018	2019
TE	0.8490	0.8191	0.7497	0.8510	0.7660
PTE	0.9000	0.8802	0.7955	0.8661	0.8556
SE	0.9433	0.9305	0.9425	0.9825	0.8952

**Fig. 1:**
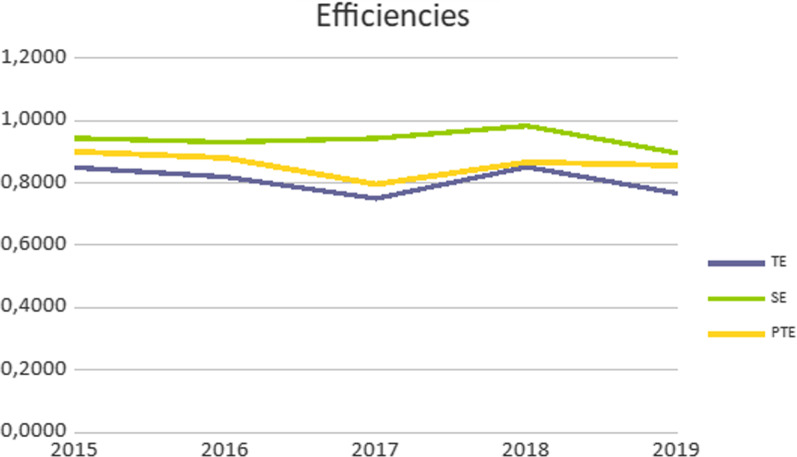
Graphical representation of the change in the efficiency values of Greek general hospitals during the study period

The results of the change in efficiency values are illustrated graphically in Fig. 1.

We observe the following regarding the change in efficiency values, based on the model used: TE follows a downward trend with fluctuations. Its value falls in 2016 and 2017 and recovers to its highest value in 2018 and falls to a low value in 2019. PE also follows a downward trend with fluctuations. Its trajectory is downward in 2016 and 2017, it recovers in 2018 and slightly decreases in 2019. On the contrary, the SE is on the rise. It rises until the year 2018 and decreases in the year 2019.

The reduction of TE with fluctuations shows the small overall reduction in efficiency in terms of PE along with SE. The reduction of PE with ups and downs shows a reduction in general of the hospital’s capacity based on the resources they use and refers to management performance. But the fluctuations refer to the relative increase in output compared to inputs. On the other hand, the increasing trend of SE shows the adequate size of hospitals in order to implement scale economies and the success of management in choosing the optimal size of each hospital.

We perform a recording and presentation of the inputs and outputs used to calculate the change in efficiency values during the study period. The presentation of these inputs and outputs is shown in Table 4.

**Table 4:** Inputs and outputs of the study during the studied period

Year	Total purchases	Number of doctors	Nursing staff	Administrative staff	Number of beds	Patients examined (TEI, TEP, former afternoons)	Number of hospitalised patients	Hospitalisation days
2015	1 709 209 870.90	10 232	30 358	6100	33 001	11 935 404	2 047 024	8 397 485
2016	1 900 755 219.39	10 748	31 172	6779	33 335	12 184 736	2 350 798	8 346 921
2017	1 995 350 198.38	11 083	31 371	6918	33 416	12 701 744	2 421 070	8 335 312
2018	2 072 526 095.37	11 328	31 072	6746	33 057	12 229 323	2 525 419	8 439 621
2019	2 312 873 216.67	11 615	31 146	7136	33 717	13 119 515	2 537 750	8 351 876

In order to estimate the relative changes of the inputs and outputs of the study, we proceeded to find and present these as a percentage change during the study period. These changes are shown in Table 5.

**Table 5:** Percentage change of the study’s inputs and outputs over the period studied

Year	Total purchases	Number of doctors	Nursing staff	Administrative staff	Number of beds	Patients examined (TEI, TEP, former afternoons)	Number of hospitalised patients	Hospitalisation days
2015	1.00	1.00	1.00	1.00	1.00	1.00	1.00	1.00
2016	1.11	1.05	1.03	1.11	1.01	1.02	1.15	0.99
2017	1.17	1.08	1.03	1.13	1.01	1.06	1.18	0.99
2018	1.21	1.11	1.02	1.11	1.00	1.02	1.23	1.01
2019	1.35	1.14	1.03	1.17	1.02	1.10	1.24	0.99

**Fig. 2:**
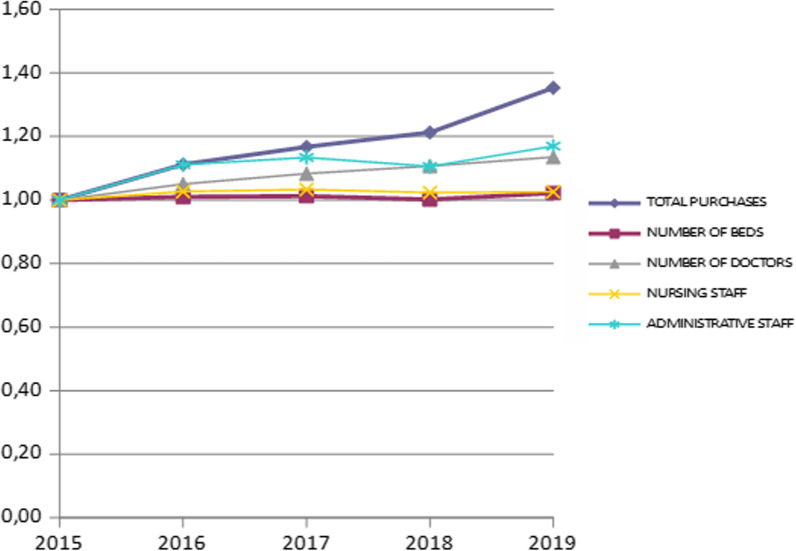
Percentage change in inputs during the study period

**Fig. 3:**
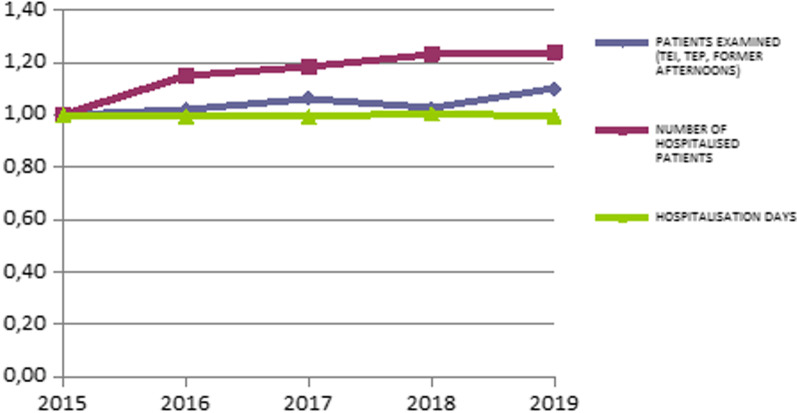
Percentage change in study’s outputs during the study period

This table very clearly shows the relative change in the inputs and outputs of the study during the period in question. Based on these values, we proceed to construct diagrams that graphically show the change of inputs and outputs. The percentage change in the inputs of the study during the study period is shown in Fig. 2, while the corresponding change for outputs is shown in Fig. 3.

From the percentage change in the inputs of the study during the given period we observe that some inputs (such as nursing staff and number of doctors) show a minimal increase. The rest of the inputs show a significant increase (markets, administrative staff, doctors). However, the increase in inputs has an unfavourable impact on efficiency.

From the percentage change of the study’s outputs, it appears that hospitalisation days show almost no change, while the patients examined, and the number of hospitalised patients show an increase. The increase in outputs predisposes a positive change in efficiency. Interventions that have contributed to the change of efficiency, were the result of specific HPs. The government SYRIZA-ANE designed the HP, the Greek MoH determined their measures. HP was implemented based on the laws of the SYRIZA-ANEL governments.

From these diagrams, the change in the study’s inputs and outputs is confirmed in a detailed and graphical way, in order to be useful for the interpretation of the change in efficiency.

Based on the literature review and Introduction analysis, the HPs of the SYRIZA-ANEL governments can be classified into the following categories and their effect on the inputs and outputs of the efficiency study can be estimated:

HPs of the SYRIZA-ANEL governments affect inputs and outputs and the change in hospital efficiency values as follows:

A) PCnH and PVQH with an impact on inputs:
  1. Policies that reduce inputs (and tend to increase efficiency):
    Changing the mode of funding through a new social security institution [39], tends to reduce the cost of health services. However, this policy alone does not reduce financial inputs, as total hospital costs increase significantly during this period. The government is in the act of developing the dual-speed PFY system [19]. This development definitely improves the overall health system by operating PFY as a filter. In this study, however, the impact on efficiency in public general hospitals (secondary health care) is examined. So, in this respect, the development of the PFY system causes a decrease in the number of staff, given that a number of staff are seconded from the PFY and that some staff are directed from general hospitals to the PFY and therefore tend to increase efficiency. At the same time, this function filters and reduces the number of incoming patients (which is analysed below).
    The ESY human resources are reorganised during this period. As a result, staff are moved from hospitals where they were in surplus to hospitals where there was a shortage. This policy addresses the needs of previously redundant personnel and has a direct impact on the change in efficiency. Governments during this period follow previous e-health development policies [40]. The further development of e-health reduces the cost of providing health services, through restrictions on visits and prescriptions.
    An effort is made to allocate staff correctly during this period. The publication of the Health Atlas for all regions of the country [41] leads to a process of recruitment which entails reduction of staff, where necessary and the proper distribution of personnel [41].
  2. The following implemented policies of the SYRIZA-ANEL governments increase inputs. This increase is made in order to offer more health services and to meet the criterion of equal access to health services, albeit that this policy tends to reduce efficiency. The policies are as follows:
    The policy of increasing health funding [42] has a slight impact on an increase in the number of staff and a higher impact on an increase in the cost of health services. Governments tried to increase funding to meet the criterion of equality in the provision of health services and to be able to offer more services to health recipients after the previous period when funding and health services had been reduced. Although the partial increase in funding, under the influence of the Memorandum, tends to increase inputs, it has a positive effect on incoming and therefore outgoing patients, as described below. In general, health policies have more than one dimension in their impact on inputs and outputs. The increase of individual categories of costs and the total costs of hospitals [43] increases the financial costs of hospitals. It is noted that the increase in funding is evident from the de facto increase in hospital expenses for the years referred to in our study and as announced on the website of MoH.
    The policy of state and fund financing [44] increases costs. The existence of closed budgets keeps costs in check but does not exclude emergency funding and additional costs if they are approved. The abolition of tickets increases the inputs used by general hospitals. The policy of expanding beneficiaries [45] increases the cost and number of doctors.
    The policy of providing extended services to beneficiaries [46] tends to increase costs (due to resources required). Policies to promote health service recipients’ rights [45, 47] slightly increase the number of staff (administrative and nursing).
B) HPs affecting outputs:
  1. Policies that increase outputs (and tend to increase efficiency)
    The SYRIZA-ANEL governments, in accordance with their electoral commitments, seek to offer more public and free health services. Although the effect of the memoranda prevents budgets from increasing, expenditure finally increases. A policy of increasing health funding [42] increases inputs as mentioned above. However, the increase in the number of services provided also causes an increase in the number of incoming patients and therefore of outpatients and hospitalisation days, so in this respect it has a positive effect on efficiency. A policy of state and fund financing is in place [44]. This policy tends to increase the number of incoming (and therefore outgoing patients) and therefore has a positive effect on efficiency.
    The policy of expanding beneficiaries [45] causes an increase in outgoing patients. The policy of expanding the provision of services to beneficiaries [46] directly causes a significant increase in the number of outgoing patients. This is part of ensuring the provision of free health services for all but is detrimental to efficiency.
    The abolition of tickets encourages recipients of health services to seek health services in a public general hospital. This reduces inputs but, on the other hand, definitely increases the number of outgoing patients and the number of hospitalisation days. The modernisation policy of Mental Health (MH) services increases the presence of health service recipients in public health units.
  2. Policies that reduce outputs (and tend to reduce efficiency)
    Development of a dual-level PFY System [19] causes a reduction in the number of recipients of health services who come to hospitals (and therefore of outgoing ones).

**Fig. 4:**
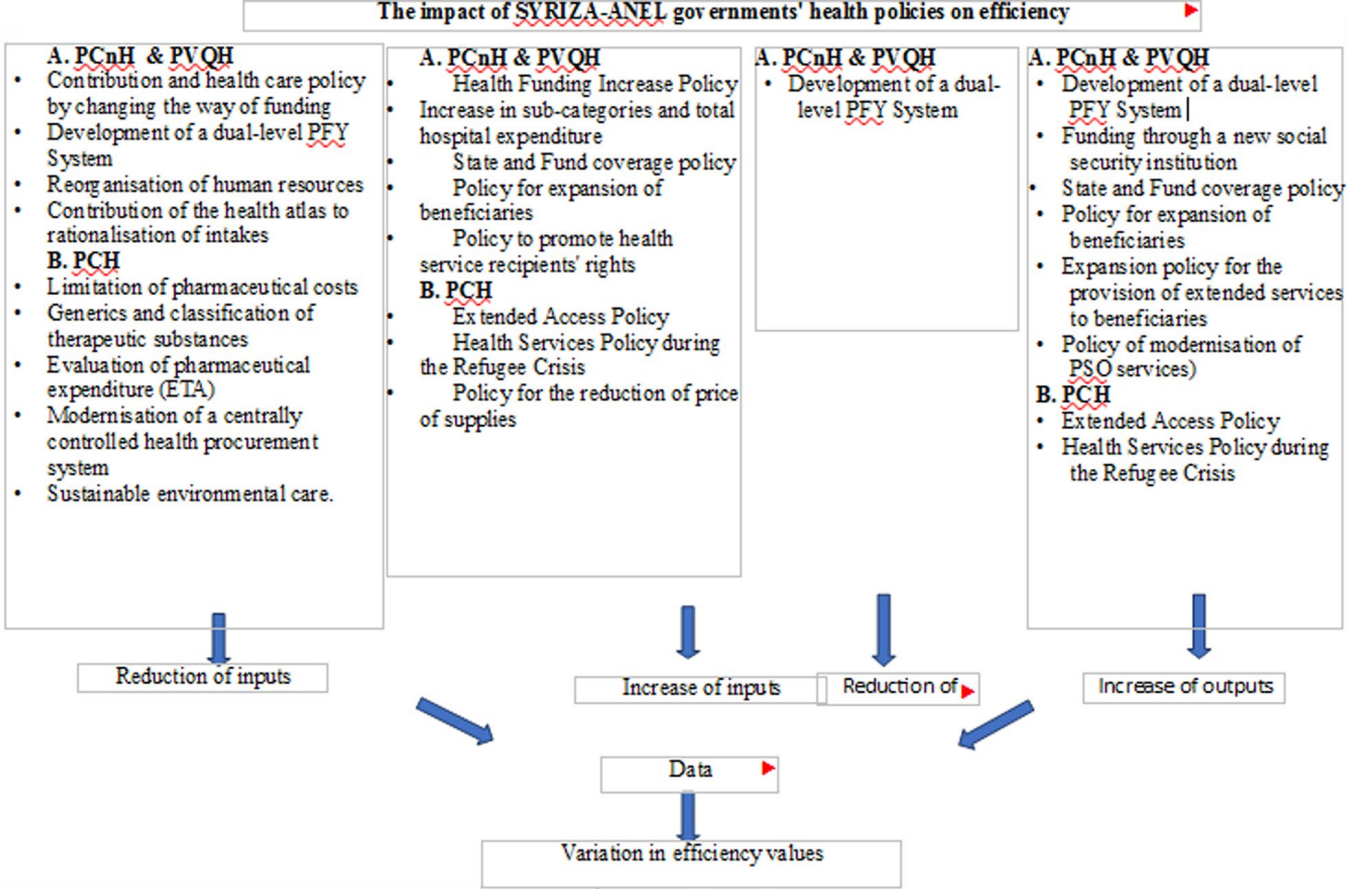
The effect of each of the health HPs on the inputs and outputs used in the efficiency study

The PCHs exercised by the Syriza-ANEL governments which affect efficiency are:

A) PCHs which reduce inputs
  Pharmaceutical cost control policy with annual budgets for classification levels of therapeutic chemical substances [48] reduces pharmaceutical costs. Pharmaceutical cost policy with drug catalogues and generics [49] reduces pharmaceutical costs.
  Health Technology Assessment (HTA) pharmaceutical expenditure policy following reconstitution of the Pharmaceutical Evaluation Committee [50, 51] causes pharmaceutical cost reduction. The modernisation policy of the centrally controlled health procurement system [19] causes the reduction of procurement costs.
  Sustainable environmental health care with simultaneous limitation of cost [52] causes energy costs to be reduced.
  PCHs that increase inputs are: the extended access policy (negative cost impact) [53] causes cost increase. The health service policy during the refugee crisis (negative impact on costs) [54], increases costs. Figure 4 shows the impact of SYRIZA-ANEL government health policies on efficiency. Table 6 shows the effect of each of the health HPs on the inputs and outputs used in the efficiency study.

**Table 6:** Effect of HPs on inputs and outputs (Authors, 2022)

Health policies	Total purchases	Number of doctors	Nursing staff	Administrative staff	Number of beds	Patients examined	Hospitalised patients	Hospitalisation days
PCnH								
Policy to increase health financing	+	+	+	+			+	+
Changing the mode of financing through a new social security institution	−							
Increase in sub-categories and total hospital expenditure	+							
Policy to cover funding from the State and Funds and not from recipients of health services	+					+	+	+
PVQH								
Development of a dual-level PFY System		−	−			−	−	−
Policies for expansion of beneficiaries	+	+	+	+			+	+
Policies for expanding services for expansion of beneficiaries–refugees	+							
Policies for the promotion of rights of health service recipients		+	+					
Policies for reorganisation of ESY human resources		−	−	−				
EHealth Development Policies	−							
Policy for the modernisation of Mental Health Services (PSY)						+		
Publication of Health Atlas for all regions of the country		−	−	−				
PCH								
Pharmaceutical cost control policies with annual budgets for classification levels of therapeutic chemical substances	−							
Pharmaceutical Cost Limitation Policies with Drug Directories, Generics, Central Procurement	−							
Pharmaceutical Expenditure Evaluation Policy with Reconstitution of the Committee for the Evaluation of Medicinal Products	−							
Cost reduction by providing annual budgets for classification levels of therapeutic chemical substances	−							
Policy for the modernisation of the centrally controlled health procurement system	−							
Environmentally sustainable health care with simultaneous limitation of costs	−							
Extended Access Policy	+							
Health ServicePolicy during the Refugee Crisis	+
  The depiction of the positive or negative impact of the HPs of the SYRIZA-ANEL governments on the inputs and outputs of efficiency shown in the table is useful for the discussion stage that follows.

### Discussion

The study raises a number of issues that deserve to be discussed further:

A) According to the Window-DEA study on the effect of the HPs of the Greek SYRIZA-ANEL governments on efficiency and on costs (costs being of significant importance to governments), the following can be noted: Regarding the change in the efficiency of Greek general hospitals, the health policies implemented during the SYRIZA-ANEL governmental period reduce the efficiency of Greek public general hospitals with ups and downs. In any case, changes in efficiency also depend on the model used, which in our case is particularly detailed, taking into account a wide range of inputs and outputs. Importantly, at this point, the following must be noted: The SYRIZA-ANEL government remained in power until July 2019 [55]. The latest values for a full year of governance refer to the year 2018. Efficiency values for the year 2019 include a six-month governmental period at least of the subsequent New Democracy government [56]. In light of this, by taking 2018 efficiency values as those of the last full year of the SYRIZA-ANEL governance, technical, pure and scale efficiency values are on the increase.
  The health policies of governments during the period under study, which for the most part falls within the context of the Greek economic crisis, aim to reduce costs only in part. Cost reduction is intended to be implemented through policies that apply technocratic measures following on from previous PCHs (such as cost constraints through further implementation of ePrescription). In this respect, health policies contribute to increased efficiency [57]. PCHs such as pharmaceutical cost containment policy with drug catalogues, generics, pharmaceutical cost control policy for classification of therapeutic substances and HTA pharmaceutical expenditure assessment policy have a direct impact on cost reduction and increase efficiency. PCnHs and PVQHs are distinguished into policies that reduce costs (and therefore increase efficiency) and policies that increase costs to achieve equality in the provision of health services (and therefore reduce efficiency).
  The SYRIZA-ANEL government reduced costs through PCnHs and PVQHs by changing the mode of funding, developing a dual-level PFY system [56], reorganising human resources and including the health atlas in the rationalisation of intakes. It also reduced costs by reducing pharmaceutical expenses through generic drugs and classification of therapeutic substances, by assessing pharmaceutical costs (HTAs), by modernising a centrally controlled health procurement system and by sustainable environmental care.
  Costs, on the other hand, increase due to certain PCnHs and PVQHs. Examples of these policies are an increase in health funding, an increase in individual categories and in total hospital costs, the policy of covering funding from the state and, the policy of expanding beneficiaries, and the policy of promoting the rights of health service recipients (minimally). Costs also rise with some of the PCHs, such as extended access policy and the health service policy during the refugee crisis [54].
  It is noted that policies are divided in terms of their impact on costs, and this concerns all three major policy categories. Policies that reduce costs (and therefore attempt to increase efficiency) and policies that increase costs (and therefore result in reduced efficiency) emerge.
  A reduction in the values of efficiency mean that the health policies failed to increase the hospital’s efficiency [58]. The reduction in the values of technical and pure efficiency means that the health policies implemented by the SYRIZA-ANEL governments failed to increase the efficiency of Greek general hospitals. The aim of health policies to achieve efficiency increase was not fulfilled. Notwithstanding this the SYRIZA-ANEL governments managed to increase the values of efficiency of scale, which is particularly important.
B) Regarding the detailed impact of the health policies of the SYRIZA-ANEL Governments on the inputs and outputs used for the study and on the efficiency of Greek general hospitals, the following can be observed:
  Government health policies aim to reduce inputs in order to increase efficiency. The study found that some of the PCnHs and PVQHs tend to reduce inputs, others to increase them, the latter having a negative effect on efficiency. The reason for the detrimental effect of inputs is that the policies mentioned are aimed at achieving equality and justice. Thus, from the study of inputs, there is a large increase in economic terms, but stability in the number of beds (given that the number of beds is the most reliable data in an attempted change). However, the picture of inputs is that of an overall increase, which explains the negative impact of health policies in terms of efficiency [59]. It seems that the increase in the number of recipients of health services, especially non-insured persons—victims of the ongoing economic crisis—and refugees, resulted in the attempted increase in efficiency being held back.
  Governments’ policies generally aim to increase outputs in order to increase efficiency [60]. Indeed, most of the applied PCnHs and PVQHs but also the PCHs, increase outputs. Funding through a new social security institution, the policy of state and fund financing, the policy of expanding beneficiaries, the policy of expanding the provision of services to beneficiaries, the policy of modernising MH services, cost policies, the policy of extensive access and the policy of providing health services during the refugee crisis, increase outputs. On the contrary, the two-speed primary health policy, although beneficial to the general health system, only reduces the outputs of general hospitals.

The change in efficiency values can be interpreted in a one and only way: that of the change in inputs and outputs over the years and the effect of HPs on this change. We can observe the following 6 points on which the discussion takes place:

1. Survey inputs remain stable or increase:
  Some of the inputs (markets) increase significantly, others (doctors, administrative staff) increase considerably, and some (nursing staff, number of beds) remain stable. This is detrimental to the rate of efficiency.
2. Survey outputs remain stable or increase:
  One of the outputs (hospitalisation days) remains stable, one (patients examined) increases with ups and downs, and one (number of hospitalised patients) increases greatly. This is conducive to an increase in efficiency [61].
3. HPs increase inputs:
  The health policies of the SYRIZA-ANEL governments are not exclusively oriented towards reducing inputs (and thus increasing efficiency). Undoubtedly, some of these policies attempt to reduce costs and inputs (or at least to have a diminishing effect and to contain their potential increase). However, other policies directly increase costs, leaving the possibility of increased purchases and an increase in the number of staff, as in the case of other countries [62]. The reason for this is that, on the one hand, the policies seek to ensure equality and fairness in access to health services for all citizens and, on the other hand, to develop the public health system which was affected by the previous economic crisis.
4. HPs increase outputs:
  Policies that ensure equality and fairness in access to health services for all citizens and the development of the public health system, which was affected by the previous economic crisis, increase outputs (number of outgoing patients, patients examined).
5. The increase in outputs is followed by the increase in inputs:
  It is noted that the government’s effort to increase outputs (which is a requirement for a government that aspires to provide increased access and equality in health services), is accompanied by an increase in inputs. Although an increase in inputs may to some extent be a desirable option for a government in order to avoid the brain-drain effect, to create jobs, or to deal with the adverse consequences of job losses in the economic crisis, it nevertheless contributes adversely to efficiency. This phenomenon (increase of outputs with simultaneous increase of inputs) as well as the reverse (decrease of inputs with simultaneous decrease of outputs) requires further investigation in order to become a general rule that can be taken into account for HPs and change in efficiency.
6. The relation of the formation of the output value to the input value leads to a change in efficiency.

## Conclusions

The HPs of the SYRIZA-ANEL Governments extend to the three major HP categories that are applied during an economic crisis. Unlike pre-existing HPs aimed at cost reduction, these policies seem to have a dual orientation: some policies reduce the cost of a category and contain the total cost, thus positively contributing to efficiency increase. Some policies are aimed more at fulfilling the criterion of equality in the provision of health services and thus the cost inevitably increases. The overall picture of inputs shows an increase. Regarding outputs, a significant increase is achieved. It is the relative change in outputs as compared to inputs that ultimately determines the change in efficiency. From the window-DEA study, three relatively “good” years emerge (2015, 2016, 2018) and two “bad years” (2017, 2019). The overall picture of values is downward, and this also depends on the nature of the model used which in our case is a very detailed one. The inputs and outputs used in DEA studies are instrumental in measuring efficiency. However, it is the relative change in outputs as compared to inputs that determines the change in efficiency. The years when outputs increase significantly as compared to the increase of inputs are observed as being more efficient and vice versa. Also, a more general pattern is confirmed: an increase in outputs requires an increase in inputs.

## Data Availability

Data is available upon request.

## Abbreviations

HP: Health policies
DEA: Data Envelopment Analysis
TE: Technical efficiency
PE: Pure (technical) efficiency
SE: Scale efficiency
DMU: Decision Making Unit
YPE: Health Regions
PCnH: Policies intended to change the level of contributions for publicly funded healthcare
PVQH: Policies intended to affect the volume and quality of publicly funded health care
PCH: Policies intended to affect the costs of publicly funded healthcare
MoH: Greek Ministry of Health
PFY: Primary Health Care
MH: Mental Health

## Declarations

### Dedication

This paper is a dedication in memory of the Professor (Nikitas-Spiros Koutsoukis) of the Department of Political Science and International Relations at the University of the Peloponnese, who passed away during the preparation of this paper.
